# Time to Surgery for Patients with Esophageal Cancer Undergoing Trimodal Therapy in Ontario: A Population-Based Cross-Sectional Study

**DOI:** 10.3390/curroncol29080466

**Published:** 2022-08-20

**Authors:** Nader M. Hanna, Paul Nguyen, Wiley Chung, Patti A. Groome

**Affiliations:** 1 Department of Surgery, Division of General Surgery, Queen’s University, 76 Stuart Street, Kingston, ON K7L 2V7, Canada; 2 Department of Public Health Sciences, Queen’s University, Kingston, ON K7L 2V7, Canada; 3 ICES Queen’s, Queen’s University, Kingston, ON K7L 2V7, Canada; 4 Department of Surgery, Division of Thoracic Surgery, Queen’s University, Kingston, ON K7L 2V7, Canada; 5 Division of Cancer Care and Epidemiology, Queen’s Cancer Research Institute, Kingston, ON K7L 2V7, Canada

**Keywords:** esophageal cancer, time to surgery, epidemiology, geographical variability, treatment interval

## Abstract

Patients with resectable esophageal cancer are recommended to undergo chemoradiotherapy before esophagectomy. A longer time to surgery (TTS) and/or time to consultation (TTC) may be associated with inferior cancer-related outcomes and heightened anxiety. Thoracic cancer surgery centers (TCSCs) oversee esophageal cancer management, but differences in TTC/TTS between centers have not yet been examined. This Ontario population-level study used linked administrative healthcare databases to investigate patients with esophageal cancer between 2013–2018, who underwent neoadjuvant chemoradiotherapy and then surgery. TTC and TTS were time from diagnosis to the first surgical consultation and then to surgery, respectively. Patients were assigned a TCSC based on the location of the surgery. Patient, disease, and diagnosing physician characteristics were investigated. Quantile regression was used to model TTS/TTC at the 50th and 90th percentiles and identify associated factors. The median TTS and TTC were 130 and 29 days, respectively. The adjusted differences between the TCSCs with the longest and shortest median TTS and TTC were 32 and 18 days, respectively. Increasing age was associated with a 16-day longer median TTS. Increasing material deprivation was associated with a 6-day longer median TTC. Significant geographic variability exists in TTS and TTC. Therefore, the investigation of TCSC characteristics is warranted. Shortening wait times may reduce patient anxiety and improve the control of esophageal cancer.

## 1. Introduction

Almost half of all patients with esophageal cancer in North America present with a disease that is unresectable [1,2]. For the remaining 50%, the time to surgery (TTS) is of the utmost importance. Previous studies have reported superior post-operative and survival outcomes with a shorter time to treatment for many cancer sites [3,4], but this remains a topic of debate in esophageal cancer [5,6,7,8]. Shorter TTS may relieve anxiety [9] for the patients and their caregivers, avoid symptom progression whilst awaiting surgery, and reduce the overall burden on healthcare systems by decreasing the number of cancer-related visits between diagnosis and treatment. Current guidelines recommend neoadjuvant chemoradiotherapy before surgical resection in operative candidates with stages Ib-IVa of the disease [10]. TTS is likely longest for this subset of esophageal cancer patients.

In Ontario, thoracic surgery services underwent a process of regionalization between 2007–2011 by creating thoracic cancer surgery centers (TCSCs) that coordinate the management of these patients [11]. This coordination is designed to improve postoperative outcomes and begins from the time of patient referral through to the postoperative follow up and cancer surveillance. Additionally, some patients are discussed at the multidisciplinary tumor board meeting. Studies investigating the effects of regionalization have demonstrated superior outcomes [12,13,14], but only one examined wait times [15] in patients with lung cancer and found no differences before and after regionalization. Such an investigation has not yet been conducted in esophageal cancer in North America.

Multiple factors may be associated with longer wait times. TTS can be further partitioned to examine the length of time to the first surgical consultation (TTC). Most patients are seen only once by a surgeon prior to their operation. Knowledge of those factors associated with esophageal cancer treatment wait times may help to identify vulnerable patient populations and uncover areas for the improvement of health services. Thus, the objectives of this study are to report the TTS and TTC in Ontario, to identify the variability between TCSCs, and to investigate factors associated with both of these wait times in a contemporary cohort of Ontario patients undergoing neoadjuvant chemoradiotherapy and surgery for esophageal cancer.

## 2. Materials and Methods

### 2.1. Study Design and Setting

This was a population-level cross-sectional study that used routinely collected healthcare administrative data housed at ICES. ICES is an independent, non-profit research institute, whose legal status under Ontario’s health information privacy law allows it to collect and analyze health care and demographic data, without consent, for health system evaluations and improvements. ICES holds numerous databases and registries. These datasets were linked using unique encoded identifiers and analyzed at ICES (Supplementary Table S1).

In Ontario (with a 14.6 million population), cancer diagnoses are prospectively registered in the Ontario Cancer Registry (OCR), which captures 96% of incident cancer diagnoses [16]. Ontario residents participate in a universal healthcare model which is free at the point of entry via the Ontario Health Insurance Plan (OHIP) and is free of charge.

### 2.2. Study Population

**Figure 1:**
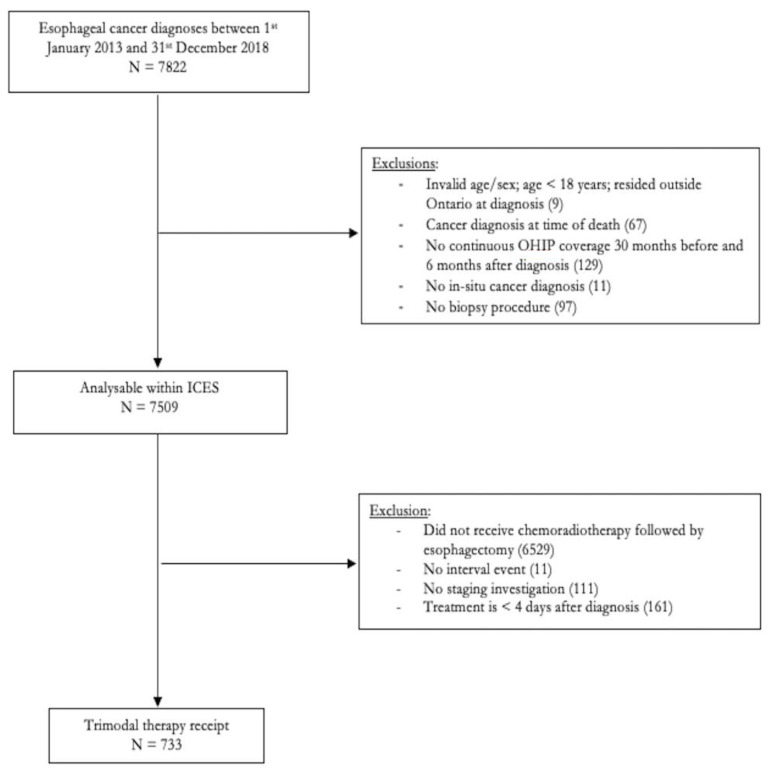
Cohort Creation.

From the OCR, we identified Ontario patients diagnosed with incident esophageal cancer (ICD-O-3 topography, codes C15 and C16) between January 2013 and December 2018, who underwent chemoradiotherapy and then surgery (Supplementary Tables S2 and S3). We used the Activity Level Reporting (ALR) database for obtaining the details on chemotherapy and radiotherapy. Exclusions included patients younger than 18 or older than 105 years of age, residing outside of Ontario at the time of diagnosis, without a diagnostic biopsy procedure, without an investigation or consultation before treatment, or with a cancer diagnosis at the time of death (Figure 1). These selection criteria were chosen to create a cohort comprised of patients that would conceivably adhere to the Cancer Care Ontario [10] pathway and be eligible for trimodal therapy. Further exclusions were based on ICES data availability.

### 2.3. Outcome Definitions

Time to surgery (TTS) and time to consultation (TTC) were calculated starting from the diagnosis. The date of diagnosis was defined as the date on which an endoscopic biopsy was obtained. The biopsy date was categorized according to whether it was within 2 weeks of the corresponding OCR date. If there was more than one biopsy date within two weeks of the OCR date, then the earliest of these was used. For patients for whom the biopsy date was more than two weeks before or after the corresponding OCR date, we instead used that OCR date as the date of diagnosis. If there was no record of a biopsy (but there was a histological diagnosis), then the OCR date was used. Dates of surgery and consultation were obtained from the OHIP physician billing database. The date of consultation refers to the first visit with a thoracic surgeon between diagnosis and surgery.

### 2.4. Covariates and Data Sources

The thoracic cancer surgery center (TCSC) is where a patient received their surgery. There are 15 designated TCSCs in Ontario [17]. These 15 TCSCs were anonymized and labelled 1–15 in our dataset. We used the INST database to identify the hospital that the surgery took place in. If it was one of the designated TCSCs, then it was assigned as such; however, if it was in an institution that was not one of the 15 TCSCs, then we labelled it as “non-TCSC.”

The Registered Persons Database was used to obtain most of the demographic data. We categorized the data according to age and sex. Comorbidity was assigned based on the Johns Hopkins Aggregate Diagnosis Groups (ADGs), diagnosed between 6 and 30 months before the cancer diagnosis date via the Discharge Abstract Database (DAD) and National Ambulatory Care Reporting System (NACRS). The ADGs were created using the Johns Hopkins ACG System v10.0.1 (build 879). Rurality was categorized into urban/rural, assigned using the Postal Code Conversion File (PCCF). Socioeconomic status was measured using area-level deprivation quintiles [18] and assigned using the PCCF and Ontario Marginalization Database. Recent immigration (IRCC Permanent Resident Database) was dichotomized into yes/no categories, the former having landed in Canada within 5 years of diagnosis. Race was unavailable in these databases. Tumor location and histology were categorized. The stage was obtained in multiple ways to ensure that it was as complete as possible. First, we used the *best_stage* variable in the OCR (which provides the pathological stage for this surgically treated group). We supplemented this information by creating a separate stage variable, according to the 8th edition of the American Joint Committee on Cancer (AJCC) staging system [19], using the individual T, N, and M categories contained in the ALR database. The diagnosing physician factors collected were primary specialty, years since graduation, and academic center affiliation, obtained from the ICES Physicians Database. We captured the use of emergency department (ED) and hospital admissions between diagnosis and the start of treatment using the DAD and NACRS. These variables were categorized as none, one, and more than one.

### 2.5. Statistical Analyses

Descriptive statistics were used to describe the cohort and the TTS and TTC in Ontario, using medians with interquartile ranges and the 90th percentile. The length and distribution of each time interval were compared between TCSCs. We performed bivariate analyses to examine associations between the covariates and outcomes using quantile regression modelling [20]. We created multivariable quantile regression models to evaluate the independence of the association between each covariate included in the model and the outcomes, whilst controlling for other covariates, except the stage and diagnosing physician factors. Finally, we performed pre-specified sensitivity analyses to assess whether the covariates associated with TTS remained significant after controlling for TTC. All analyses were performed at ICES Queen’s using the SAS software v9.4 (SAS Institute Inc., Cary, NC, USA). All data were stored, accessed, and analyzed at ICES Queen’s. In accordance with ICES policy, data on < 6 patients were suppressed to prevent patient identification.

## 3. Results

We identified 7822 patients diagnosed with esophageal cancer between January 2013 and December 2018. After the exclusions, the final cohort comprised of 733 patients (Figure 1). Most (40.1%) were aged 60–69 years, male (79.4%), had a total ADG between 4–6 (33.2%), were non-immigrants (93.9%), living in urban locations (83.8%), and had tumors in the lower esophagus (57.7%) which were adenocarcinoma (78.9%). Ten patients (1.4%) had surgery in a non-TCSC hospital. The number of patients receiving trimodal therapy increased from 87 in 2013 to 153 in 2018. Stage data were missing for 38% of patients. Gastroenterologists (37.8%) and general surgeons (36.8%) diagnosed a similar proportion of patients. In total, 21.7% of the diagnosing physicians were affiliated with an academic center (Table 1).

### 3.1. Length of Time Intervals

**Figure 2:**
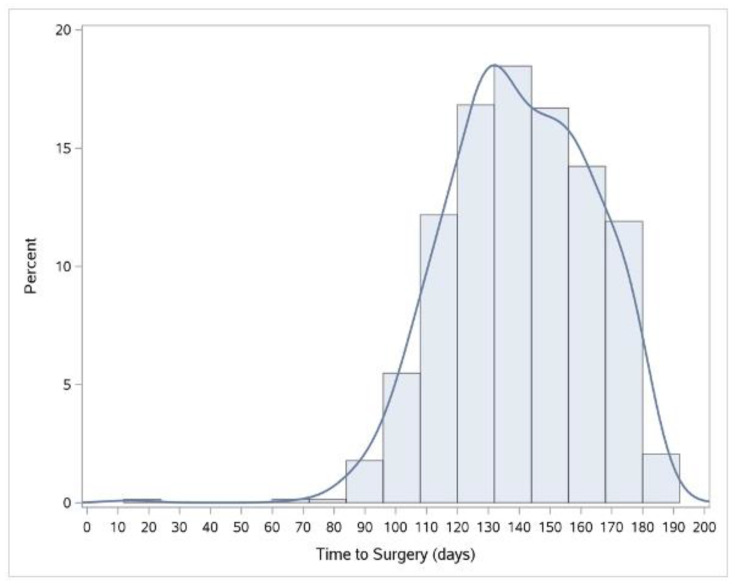
Bar chart depicting the distribution of the time to surgery for esophageal cancer in Ontario between 2013 and 2018.

**Figure 3:**
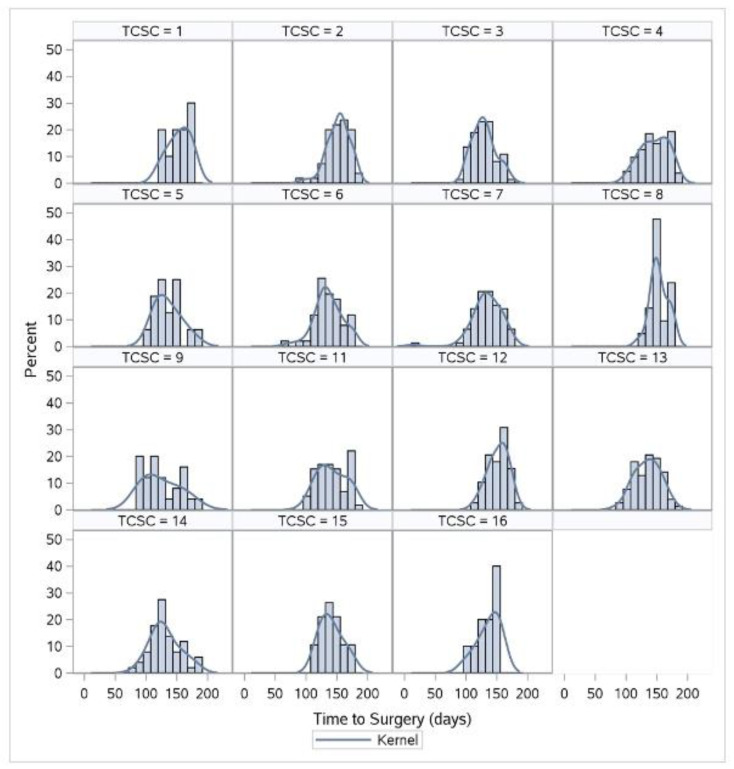
Bar chart demonstrating the comparison of the time to surgery for esophageal cancer distribution amongst thoracic cancer surgery centers in Ontario between 2013 and 2018.

The median TTS was 140 days (IQR: 125–158 days) and the 90th percentile was 171 days (Figure 2). The median and 90th TTC were 14 (IQR: 6–26 days) and 41 days, respectively. Table 2 reports the TCSCs’ TTSs and TTCs in days at the 50th and 90th percentiles. The shortest median TTS was 125 days (IQR 119–133) and the longest was 156 days (IQR: 141–164). At the 90th percentile, the shortest and longest TTS were 154 and 176 days. The shortest median TTC was 8 days (IQR 2–21 days) and the longest was 27 days (IQR: 20–36). The shortest and longest 90th percentile TTC were 18 days and 148 days, respectively. Figure 3 illustrates the TTS variability both amongst the TCSCs and within each TCSC. Compared to the provincial distribution, TCSCs 8 and 16 demonstrated less variability within their centers. Conversely, TCSC 4, 9, and 11 demonstrated the greatest variability within each individual center.

### 3.2. Changes in TTS and TTC Lengths by TCSC after Adjusting for Covariates

Statistically significant differences in the TTS and TTC remained amongst the TCSCs in the adjusted analyses, but controlling for the confounding by the study covariates changed some of the estimates (Table 3 and Table 4). The biggest change was in TCSC 9, where the -22-day unadjusted difference compared to the referent group was reduced to -13 days. TCSC 5 had a 5-day longer median TTS after adjustment. The other TCSCs demonstrated changes of 4 days or less.

TCSCs 8, 9, and the non-TCSC demonstrated a 4-day change in the median TTC after adjustment. The other TCSCs demonstrated a change of 3 days or less. At the 90th percentile, the biggest change was seen in TCSC 12, with a 22-day shorter TTC (21 days to -1 days longer than the referent group), followed by the non-TCSC, with a 20-day shorter TTC after adjustment (99 days to 79 days longer than the referent group).

### 3.3. Bivariate Analysis of Factors Associated with TTS and TTC Lengths

Table 2 reports the TTS and TTC according to the covariates. Increasing age was associated with a longer TTS, but not TTC. Those aged 18–49 years had a shorter median TTS compared to those aged 70+ (131 vs. 144 days). Those in the highest deprivation quintile had a longer TTS (146 days (IQR 127–160)) than those in the lowest deprivation (134 days (IQR: 121–154)) (*p* = 0.03), but there was no difference in TTC. Patients in rural locations had a longer TTS than those in urban areas (145 versus 138 days, *p* = 0.04), but there was no statistical difference in TTC (*p* = 0.36). There were no statistically significant differences in TTS or TTC regarding sex, comorbidity burden, or immigration status.

Tumor location was significantly associated with the median TTS. Those in the ‘other’ category waited a median time of 131 days (IQR: 128–161) compared to those with tumors in the lower esophagus (141 days (IQR: 125–156)). This was not significant at the 90th percentile. Tumor location was not associated with the median TTC but was significant at the 90th percentile, with a 30-day difference (*p* < 0.0001) between those in the ‘other’ category (23 days) compared to those in the middle esophagus (53 days). Histology was not associated with either TTS or TTC at the median or the 90th percentile. Those with a stage-IV cancer waited 4 days less for a consultation at the median than those with a stage-I or -II cancer (12 vs. 16 days; *p* = 0.006). Stage was not significantly associated with the 90th percentile TTC.

There were no diagnosing physician characteristics associated with the median or 90th percentile TTS. Patients whose tumors were diagnosed by a thoracic surgeon had the shortest TTC at the median (-4 days (IQR -9–0)) and 90th percentile compared to those diagnosed by physicians of other specialties. Patients diagnosed by a physician with an academic affiliation had a shorter TTC at the median (6 versus 14 days; *p* < 0.0001) and 90th percentile (29 vs. 36 days; *p* = 0.03). Patients diagnosed by a physician with 30+ years in practice waited 11 days longer for consultation than those diagnosed by a physician who had been in practice between 1–9 years (*p* = 0.02).

Patients who had more than one hospital admission between diagnosis and first treatment had a statistically longer median TTS (151 vs. 138 days; *p* < 0.0001) but a shorter median TTC compared to those without (9 versus 15; *p* = 0.004). There was no difference in TTS or TTC at either the median or the 90th percentile in patients who had one or more ED visits compared to those who did not.

### 3.4. Factors Associated with TTS and TTC

In the adjusted analyses (Table 3), the only patient characteristic associated with the median TTS was age. Compared to those aged 60–69 years, younger patients (18–49 years) had an 11-day shorter TTS, while those aged 70+ had a 4-day longer TTS (*p* = 0.01). This was not statistically significant at the 90th percentile. Neither sex, comorbidity, deprivation, rurality, immigration status, nor disease characteristics were associated with a prolonged TTS at either the 50th or 90th percentiles. The only factor significantly associated with the median TTC was the level of material deprivation. Those who lived in more deprived areas waited 6 days longer than those in the least deprived areas (Q5 = 4 (CI: 1–7) days versus Q2 = −2 (CI: -6–1 days)) (Table 4).

### 3.5. Sensitivity Analysis

After controlling for TTC, age and TCSC remained factors that were significantly associated with the median TTS. At the 90th percentile, TCSC was no longer significant. The factor that became significantly associated with TTS after controlling for TTC was material deprivation at the 50th percentile (Supplementary Table S4).

## 4. Discussion

This study found that the median TTS for the whole province was 140 days and the median TTC was 14 days. The difference between the TCSCs with the longest and shortest TTS was 42 days at the 50th, and 21 days at the 90th percentile. After adjusting for the relevant covariates, TCSC 9 had a 9-day longer median TTS. Other TCSCs varied by 5 days or less, suggesting that there was minimal confounding by the other covariates in those TCSCs. Additionally, older patients (70+ years old) waited 16 days longer for surgery compared to younger patients (18–49 years old). 

TTC also varied significantly amongst the TCSCs, with a difference of 19 and 130 days at the median and 90th percentile, respectively, between the TCSCs with the longest and shortest TTC. The 22-day change in TTC between the unadjusted and adjusted models at the 90th percentile suggests that some confounding was present at this extreme end of the distribution. 

### 4.1. Time to Surgery in Ontario

Our median TTS of 140 days is longer than the only other population-level study performed on a Canadian cohort comprised only of esophageal cancer patients [2]. That study found that patients who underwent esophagectomy without neoadjuvant chemoradiotherapy waited a median of 114 days, but they did not report the time to surgery in patients undergoing neoadjuvant therapy. We chose to restrict our study population to those undergoing neoadjuvant chemoradiotherapy followed by surgery for three reasons. Firstly, this method creates a homogenous group of patients experiencing the same treatment in the same order, thereby reducing confounding by indication. Secondly, this group of patients has not yet been studied in isolation. They represent a typical patient that presents with curable esophageal disease, and thus warrant their own investigation. Thirdly, it is likely that the vast majority of patients in this group have stage-II or -III cancer, which partly compensates for a larger proportion of the population having missing stage data. According to the Health Cancer Care Ontario guidelines [10], those with stage-Ia cancer can be offered endoscopic resection, those with stage-IVb should not be offered surgical resection, and the remainder can be offered neoadjuvant chemoradiotherapy before surgery as per the CROSS protocol [21]. This protocol recommends five weeks of chemotherapy and 23 fractions of radiotherapy (5 factions per week), both starting on the same day. An interval of 6–8 weeks between chemoradiotherapy and surgery is recommended. Therefore, the natural wait time of at least 11 weeks (77 days) between the start of neoadjuvant treatment and surgery contributes to a large proportion of the overall TTS. 

### 4.2. Comparison between TCSCs 

To our knowledge, no other study has examined the variation in TTS between TCSCs in Ontario for esophageal cancer. Shakeel and colleagues [15] compared wait times for lung cancer before, immediately after, and five years after regionalization and did not identify a significant difference in wait times between the three groups. Since the regionalization of thoracic services implemented between 2005–2011 [11,22], several studies have sought to identify whether regionalization is associated with fewer postoperative outcomes and improved survival [12,13,14]. These studies have noted that outcomes changed very little before and after the regionalization process, and some [23] attribute this finding to the fact that there was only a modest change in the volume of resections performed per hospital. Although true for lung surgery [12,14], this volume stability is not the case for esophageal cancer surgery [13], where it was noted that the regionalization process resulted in fewer hospitals performing more operations. Of note, the change in the volume of esophageal operations has been associated with lower in-hospital mortality [13]. The standards for thoracic surgical oncology [11] (against which a hospital may be designated as a TCSC) are not exhaustive, thereby leaving room for differences in hospital resources, the presence of nurse navigators, availability of imaging modalities, and number of thoracic surgeons between TCSCs. These differences may partly explain the significant variation in TTS and TTC from one TCSC to another.

### 4.3. Factors Associated with TTS and TTC

Age was the only factor associated with a longer TTS. Patients aged 70+ years old waited almost 16 days more than those aged 18–49 years old, even after controlling for comorbid disease burden. This may be explained by the fact that older people require more pre-operative investigations than younger patients, despite undergoing the same treatment [24], which may delay the start of treatment, and therefore the surgery date, until the results of these investigations are obtained and acted upon. Our results agree with other Canadian studies that have demonstrated an association between increasing age and longer wait times for cancer treatment [25,26,27].

Interestingly, rurality was not significantly associated with a longer TTS or TTC. One unintended consequence of regionalization is that patients may be required to travel further than they otherwise would have to attend consultations and have their surgery [23]. Other cancer sites that have undergone a similar regionalization process include hepatobiliary surgery and gastric cancer surgery [28], but to our knowledge, there have been no studies examining the effects of regionalization on wait times in those patients. 

The only factor associated with a statistically significant difference in TTC was deprivation. Socio-economic differences may indicate health system structural inequities and/or access barriers. Additionally, persons living in materially deprived areas are more likely to have jobs that do not allow them to take time off work and face other personal barriers to seeking health care [29,30].

### 4.4. Strengths and Limitations

This is the first Canadian population-level study to examine the time from diagnosis to surgery in a group of homogenous patients undergoing neoadjuvant chemoradiotherapy followed by esophagectomy. Previous studies have either investigated the time to surgery in a heterogeneous cohort of patients without stratifying by the cancer site [31] or did not assess TTS variation across centers in Ontario [2]. Our study used population-level databases within the largest province of Canada, which allowed us to study many patient-, disease-, and diagnosing physician-level factors, thereby providing a broad description of the consultation and surgical wait time variations. This study incorporated some methodologic refinements, including our treatment of the diagnosis date (the beginning of each time interval). Our approach agrees with national efforts to standardize these time intervals [32]. We investigated the TTC, including the study of factors associated with TTC, and the contribution of TTC to the TTS, which is a unique addition to the literature on esophageal cancer wait times. Lastly, these results are generalizable to other healthcare settings where individual institutions are responsible for esophageal cancer management.

The stage was missing for 38% of patients, despite supplementing this variable by using a combination of individual T, N, and M categories. By restricting our study population to those undergoing neoadjuvant chemoradiotherapy followed by esophagectomy, we ensured that most patients had stage-II–III disease. Among those patients in our study with stage data, only 8% had stage I and 3% had stage IV. We therefore excluded stage from the regression analyses, which may have resulted in uncontrolled confounding between each covariate and the TTS, given that the bivariate analyses demonstrated a statistically significant association between the stage and TTS at the 50th percentile. We also did not include diagnosing physician characteristics in the regression models because of the missing data. Patients diagnosed by a thoracic surgeon may not necessarily have another consultation before treatment, and so those patients would have a TTC of zero. For those patients, TTS will likely be shorter than it is for others. TCSC-level factors, such as the annual case volume, number of surgeons, presence of a nurse navigator, and size of the catchment area, were not available in the existing databases. TCSCs with a nurse navigator may have shorter intervals. A further understanding of TCSC wait time variations is warranted. 

## 5. Conclusions

In this population-level study, we have identified a difference of 32 days between the TCSCs with the longest and shortest times to surgery. Older patients were at a greater risk of longer wait times. The results of our study can be used by several groups: clinicians and healthcare providers should be aware that older patients may require more support to navigate the system and may wish to flag these patients to the multidisciplinary oncology team as a vulnerable group; individual institutions can compare their data with the provincial median and perform an internal investigation into the potential reasons for a prolonged TTS. Future studies on institution-level characteristics associated with wait times will provide further insights into potentially modifiable factors.

## Figures and Tables

**Table 1:** Patient, disease, diagnosing physician, and healthcare system characteristics of Ontario patients undergoing trimodal treatment for esophageal cancer between 2013 and 2018.

Cohort Characteristics	Number of Patients (%)
**Age Group (Years)**	
18–49	49 (6.7)
50–59	200 (27.3)
60–69	299 (40.8)
70–79	166 (22.7)
80+	19 (2.6)
**Sex**	
F	151 (20.6)
M	582 (79.4)
**Sum of Minor ADGs**	
0	48 (6.6)
1–2	161 (22.0)
3–4	195 (26.6)
5–6	168 (22.9)
7+	161 (22.0)
**Sum of Major ADGs**	
0	334 (45.6)
1	222 (30.3)
2	121 (16.5)
3+	56 (7.6)
**Total Number of ADGs**	
0	46 (6.3)
1–3	202 (27.6)
4–6	243 (33.2)
7–9	162 (22.1)
10+	80 (10.9)
**Recent Immigration**	
No	688 (93.9)
Yes	45 (6.1)
**Material Deprivation**	
Least Deprived	154 (21.0)
2	153 (20.9)
3	138 (18.8)
4	139 (19.0)
Most Deprived	144 (19.7)
Unknown	<6
**Rurality**	
Rural	119 (16.2)
Urban	611 (83.4)
Unknown	<6
**Calendar Year of Diagnosis**	
2013	87 (11.9)
2014	115 (15.7)
2015	118 (16.1)
2016	123 (16.8)
2017	137 (18.7)
2018	153 (20.9)
**Histology**	
Adenocarcinoma	578 (78.9)
Squamous Cell Carcinoma	122 (6.6)
Other	33 (4.5)
**Tumor Site**	
Cervical Esophagus	<6
Upper Esophagus	<6
Middle Esophagus	64 (8.7)
Lower Esophagus	423 (57.7)
Gastroesophageal Junction	223 (30.4)
Other	18 (2.5)
**Stage**	
I	58 (7.9)
II	165 (22.5)
III	208 (28.4)
IV	21 (2.9)
Unknown	281 (38.3)
**Diagnosing Physician Main Specialty**	
Gastroenterology	277 (37.8)
General Surgery	270 (36.8)
Thoracic Surgery	84 (11.5)
Other	70 (9.6)
Unknown	32 (4.4)
**Diagnosing Physician Years in Practice**	
1–9	31 (4.2)
10–14	115 (15.7)
15–19	107 (14.6)
20–24	61 (8.3)
25–29	49 (6.7)
30+	66 (9.0)
Unknown	304 (41.5)
**Diagnosing Physician Academic Affiliation**	
0	441 (60.2)
1	159 (21.7)
Unknown	133 (18.1)
**TCSC Where Surgery Took Place**	
1	20 (2.7)
2	55 (7.5)
3	74 (10.1)
4	135 (18.4)
5	16 (2.2)
6	51 (7.0)
7	78 (10.6)
8	21 (2.9)
9	25 (3.4)
10	<6
11	59 (8.1)
12	39 (5.3)
13	78 (10.6)
14	51 (7.0)
15	19 (2.6)
16	10 (1.4)
17	<6
**Emergency Department Visits**	
0	652 (89.2)
1	69 (9.4)
>1	10 (1.4)
**Hospital Admission**	
0	623 (85.2)
1	96 (13.1)
>1	12 (1.6)

ADG = aggregate diagnosis groups; TCSC = thoracic cancer surgery center.

**Table 2:** Time to surgical consult and time to surgery (days) at the 50th and 90th percentiles according to the categories of associated factors in Ontario patients with esophageal cancer between 2013 and 2018.

Variable	Time to Consult (days)		Time to Surgery (days)	
	Median (IQR)	90th	Median (IQR)	90th
**Whole Cohort**	14 (6, 26)	41	140 (125, 158)	171
**TCSC**	***p* < 0.0001**	***p* < 0.0001**	***p* < 0.0001**	***p* < 0.0001**
1	9 (2, 14)	18	148 (137, 163)	170
2	11 (3, 19)	29	154 (141, 163)	175
3	16 (8, 28)	52	127 (116, 139)	156
4	8 (2, 21)	41	151 (131, 166)	174
5	10 (1, 16)	84	125 (119, 133)	154
6	12 (4, 21)	35	133 (122, 150)	175
7	16 (7, 28)	41	134 (119, 152)	162
8	10 (0, 27)	120	149 (141, 166)	171
9	11 (4, 27)	82	130 (108, 158)	176
10 *	-	-	-	-
11	17 (12, 23)	35	139 (120, 161)	176
12	27 (20, 36)	119	156 (141, 164)	171
13	27 (14, 35)	44	138 (119, 150)	161
14	10 (3, 18)	27	126 (113, 139)	162
15	13 (4, 20)	24	139 (125, 147)	169
16	23 (4, 88)	148	143 (127, 153)	155
**Age**	***p* = 0.35**	***p* = 0.28**	***p* = 0.009**	***p* = 0.55**
18–49	11 (5, 21)	41	131 (118, 154)	173
50–59	14 (6, 26)	39	137 (125, 157)	169
60–69	14 (6, 25)	36	139 (124, 156)	171
70+	16 (7, 27)	47	144 (128, 160)	173
**Sex**	***p* = 0.10**	***p* = 0.07**	***p* = 1.00**	***p* = 0.70**
F	17 (6, 27)	52	141 (125, 160)	173
M	14 (6, 26)	39	139 (125, 156)	171
**Sum of Minor ADGs**	***p* = 0.31**	***p* = 0.42**	***p* = 0.70**	***p* = 0.37**
0	18 (3, 34)	52	135 (116, 154)	173
1–2	16 (7, 26)	41	141 (124, 161)	172
3–4	13 (5, 21)	36	138 (124, 154)	169
5–6	16 (7, 27)	36	143 (126, 158)	167
7+	13 (3, 27)	40	139 (127, 159)	173
**Sum of Major ADGs**	***p* = 0.10**	***p* = 0.92**	***p* = 0.08**	***p* = 0.78**
0	14 (6, 26)	42	135 (121, 154)	169
1	14 (5, 23)	42	141 (126, 160)	171
2	14 (6, 23)	36	143 (127, 159)	172
3+	20 (5, 30)	42	143 (126, 161)	175
**Recent Immigration**	***p* = 0.80**	***p* = 0.97**	***p* = 0.42**	***p* = 0.87**
0	14 (6, 26)	41	140 (125, 157)	171
1	12 (0, 21)	41	146 (120, 163)	177
**Material Deprivation**	***p* = 0.05**	***p* = 0.95**	***p* = 0.04**	***p* = 0.55**
Least Deprived	13 (5, 25)	42	134 (121, 154)	173
2	14 (5, 25)	36	142 (127, 155)	168
3	11 (2, 24)	37	139 (123, 156)	171
4	14 (7, 24)	38	143 (125, 158)	172
Most Deprived	19 (7, 28)	45	146 (127, 160)	171
**Rurality**	***p* = 0.38**	***p* = 0.44**	***p* = 0.03**	***p* = 0.19**
Rural	16 (7, 29)	47	145 (132, 160)	174
Urban	14 (5, 25)	40	138 (123, 156)	171
**Calendar Year of Diagnosis**	***p* = 0.26**	***p* = 0.91**	***p* < 0.0001**	***p* = 0.0001**
2013	15 (4, 23)	39	134 (124, 153)	161
2014	13 (5, 21)	38	131 (118,154)	171
2015	15 (6, 27)	44	143 (129, 161)	169
2016	14 (2, 29)	41	142 (125, 162)	175
2017	18 (8, 28)	47	150 (131, 163)	175
2018	12 (6, 22)	35	135 (120, 152)	163
**Histology**	***p* = 0.89**	***p* = 0.37**	***p* = 0.32**	***p* = 0.77**
Adenocarcinoma	14 (6, 26)	40	140 (125, 159)	170
Squamous Cell Carcinoma	14 (2, 23)	53	138 (123, 154)	175
Other	14 (7, 28)	61	139 (123, 161)	175
**Tumor site**	***p* = 0.30**	***p* = 0.08**	***p* = 0.74**	***p* = 0.85**
Middle Esophagus	15 (2, 27)	53	141 (123, 156)	174
Lower Esophagus	14 (6, 26)	40	141 (125, 156)	170
Gastroesophageal Junction	15 (7, 28)	44	139 (121, 159)	171
Other	11 (4, 17)	23	131 (128, 161)	169
**Stage**	***p* = 0.0002**	***p* = 1.00**	***p* = 0.59**	***p* = 0.75**
I	16 (8, 21)	30	140 (126, 161)	170
II	16 (6, 28)	39	143 (123, 158)	174
III	13 (4, 23)	41	137 (121, 154)	165
IV	12 (0, 36)	42	155 (128, 165)	176
Unknown	14 (6, 26)	41	139 (125, 160)	171
**Specialty**	***p* < 0.0001**	***p* = 0.0001**	***p* = 0.11**	***p* = 0.35**
Gastroenterology	16 (7, 28)	46	137 (124, 156)	172
General Surgery	19 (11, 27)	40	144 (128, 159)	169
Thoracic Surgery	−4 (−9, 0)	11	133 (111, 149)	166
Other	14 (6, 23)	35	140 (120, 162)	174
**Years in Practice**	***p* = 0.19**	***p* = 0.95**	***p* = 0.26**	***p* = 0.76**
1–9	10 (1, 18)	23	131 (124, 153)	166
10–14	12 (3, 22)	39	133 (125, 155)	173
15–19	15 (7, 28)	52	144 (130, 159)	167
20–24	17 (6, 34)	49	135 (117, 157)	171
25–29	13 (4, 29)	52	138 (119, 161)	169
30+	21 (14, 31)	47	141 (130, 157)	172
**Academic Affiliation**	***p* < 0.0001**	***p* = 0.007**	***p* = 0.09**	***p* = 0.78**
No	14 (5, 24)	36	144 (125, 159)	171
Yes	6 (-2, 19)	29	134 (118, 155)	172
**ED visit**	***p* = 0.47**	***p* = 0.58**	***p* = 0.13**	***p* = 0.95**
0	14 (5, 25)	39	139 (124, 158)	171
1	20 (8, 36)	57	145 (131, 155)	171
>1	14 (11, 34)	42	150 (138, 158)	170
**Hospital Admission**	***p* = 0.16**	***p* = 0.87**	***p* < 0.0001**	***p* = 0.74**
0	15 (6, 26)	40	138 (124, 155)	170
1	13 (2, 26)	44	153 (130, 163)	172
>1	9 (4, 18)	37	151 (132, 169)	174

* There were no observations in TCSC 10 during the study period from 2013–2018. (IQR = Interquartile Range; TCSC = Thoracic Cancer Surgery Centre; ADG = aggregate diagnosis groups; ED = emergency department.)

**Table 3:** Unadjusted and adjusted differences in **TTS** (days) at the 50th and 90th percentiles according to the factor categories in Ontario patients undergoing trimodal treatment for esophageal cancer between 2013 and 2018.

Variable	50th Percentile		90th Percentile	
	Unadjusted Difference (95% CI)	Adjusted Difference (95% CI)	Unadjusted Difference (95% CI)	Adjusted Difference (95% CI)
*Adjusted Intercept*		*130 (120, 140)*		*163 (151, 174)*
**Age Group (Years)**	***p* = 0.005**	***p* = 0.01**	***p* = 0.54**	***p* = 0.35**
*Unadjusted Intercept*	*139 (136, 142)*		*172 (169, 175)*	
18–49	−11 (−19, −3)	−11 (−19, −2)	−3 (−16, 10)	−8 (−22, 6)
50–59	−1 (−10, 8)	−1 (−6, 4)	−3 (−9, 3)	−4 (−10, 1)
60–69	Ref	Ref	Ref	Ref
70+	5 (−1, 11)	4 (−1, 9)	1 (−4, 6)	−2 (−7, 2)
**Sex**	***p* = 1.00**	***p* = 0.38**	***p* = 0.69**	***p* = 0.78**
*Unadjusted Intercept*	*140 (134, 146)*		*173 (169, 177)*	
Female	Ref	Ref	Ref	Ref
Male	0 (−7, 7)	−2 (−6, 2)	−1 (−6, 4)	−1 (−7, 5)
**Sum of Minor ADGs**	***p* = 0.68**	***p* = 0.40**	***p* = 0.36**	***p* = 0.86**
*Unadjusted Intercept*	*140 (135, 145)*		*174 (170, 178)*	
0–2	Ref	Ref	Ref	Ref
3–4	−2 (−9, 5)	−3 (−9, 2)	−5 (−11, 1)	−2 (−8, 4)
5–6	3 (−5, 11)	1 (−4, 6)	−4 (−11, 3)	−2 (−8, 4)
7+	−1 (−9, 7)	−2 (−8, 4)	−1 (−6, 4)	−2 (−8, 4)
**Sum of Major ADGs**	***p* = 0.09**	***p* = 0.41**	***p* = 0.79**	***p* = 0.61**
*Unadjusted Intercept*	*135 (131, 139)*		*171 (168, 174)*	
0	Ref	Ref	Ref	Ref
1	6 (0, 12)	1 (−4, 5)	0 (−6, 6)	3 (−2, 8)
2	8 (0, 16)	4 (−1, 9)	3 (−3, 9)	3 (−4, 9)
3+	8 (−2, 18)	4 (−4, 11)	2 (−6, 10)	3 (−5, 12)
**Material Deprivation**	***p* = 0.04**	***p* = 0.32**	***p* = 0.56**	***p* = 0.21**
*Unadjusted Intercept*	*133 (128, 138)*		*173 (169, 177)*	
Least Deprived	Ref	Ref	Ref	Ref
2	10 (3, 17)	5 (−1, 12)	−5 (−12, 2)	−1 (−7, 5)
3	5 (−3, 13)	4 (−2, 10)	−2 (−8, 4)	1 (−5, 7)
4	10 (2, 18)	7 (1, 14)	0 (−6, 6)	1 (−6, 7)
Most Deprived	8 (−1, 17)	7 (0, 14)	1 (−6, 8)	2 (−5, 9)
**Rurality**	***p* = 0.05**	***p* = 0.68**	***p* = 0.17**	***p* = 0.25**
*Unadjusted Intercept*	*138 (135, 141)*		*171 (169, 173)*	
Urban	Ref	Ref	Ref	Ref
Rural	7 (0, 14)	3 (−3, 8)	4 (−2, 10)	3 (−2, 9)
**Recent Immigration**	***p* = 0.42**	***p* = 0.19**	***p* = 0.88**	***p* = 0.92**
*Unadjusted Intercept*	*140 (138, 142)*		*172 (170, 174)*	
No	Ref	Ref	Ref	Ref
Yes	−6 (−21, 9)	2 (−8, 12)	1 (−12, 14)	8 (−6, 22)
**Histology**	***p* = 0.30**	***p* = 0.06**	***p* = 0.76**	***p* = 0.72**
*Unadjusted Intercept*	*140 (137, 143)*		*171 (168, 174)*	
Adenocarcinoma	Ref	Ref	Ref	Ref
Squamous Cell Carcinoma	−4 (−10, 2)	−5 (−11, 1)	2 (−6, 10)	3 (−4, 9)
Other	4 (−7, 15)	10 (−3, 22)	4 (−10, 18)	2 (−12, 17)
**Tumor location**	***p* = 0.77**	***p* = 0.68**	***p* = 0.86**	***p* = 0.93**
*Unadjusted Intercept*	*139 (133, 145)*		*173 (169, 177)*	
Middle Esophagus	1 (−11, 13)	4 (−6, 15)	0 (−8, 8)	1 (−8, 10)
Lower Esophagus	1 (−5, 7)	0 (−4, 4)	−2 (−7, 3)	0 (−5, 4)
Gastroesophageal Junction	Ref	Ref	Ref	Ref
Other	−8 (−27, 11)	−6 (−22, 10)	−5 (−27, 17)	−4 (−20, 12)
**TCSC**	***p* < 0.0001**	***p* < 0.0001**	***p* < 0.0001**	***p* = 0.003**
*Unadjusted Intercept*	*135 (129, 141)*		*163 (156, 170)*	
01	19 (4, 34)	19 (3, 35)	11 (−5, 27)	13 (−6, 32)
02	19 (12, 26)	19 (11, 28)	13 (5, 21)	13 (3, 22)
03	−8 (−16, 0)	−5 (−12, 3)	−3 (−14, 8)	−4 (−16, 8)
04	14 (4, 24)	14 (5, 22)	12 (5, 19)	13 (5, 21)
05	−3 (−22, 16)	−8 (−28, 12)	7 (−28, 43)	7 (−19, 33)
06	−2 (−10, 6)	5 (−4, 14)	8 (−8, 24)	7 (−8, 23)
07	0 (−9, 9)	3 (−5, 11)	−1 (−10, 8)	1 (−10, 11)
08	17 (8, 26)	16 (5, 28)	11 (−14, 36)	11 (−10, 32)
09	−22 (−44, 1)	−13 (−33, 7)	4 (−20, 28)	4 (−22, 30)
10 *	-	-	-	-
11	4 (−8, 16)	4 (−8, 16)	13 (6, 20)	12 (3, 21)
12	20 (10, 30)	19 (10, 27)	10 (−2, 22)	10 (−1, 21)
13	Ref	Ref	Ref	Ref
14	−8 (−16, 0)	−4 (−13, 4)	0 (−18, 18)	0 (−17, 17)
15	4 (−9, 17)	7 (−7, 21)	6 (−17, 29)	9 (−13, 31)
Non−TCSC	8 (−14, 30)	7 (−12, 26)	−8 (−88, 72)	−9 (−64, 47)

* There were no observations in TCSC 10 during the study period from 2013–2018. (CI = confidence interval; ADG = aggregate diagnosis groups; TCSC = thoracic cancer surgery center.)

**Table 4:** Unadjusted and adjusted differences in **TTC** (days) at the 50th and 90th percentiles according to the factor categories in Ontario patients undergoing trimodal treatment for esophageal cancer between 2013 and 2018.

	50th Percentile		90th Percentile	
Variable	Unadjusted Difference (95% CI)	Adjusted Difference (95% CI)	Unadjusted Difference (95% CI)	Adjusted Difference (95% CI)
*Adjusted Intercept*		*29 (24, 35)*		*54 (35, 72)*
**Age**	***p* = 0.36**	***p* = 0.54**	***p* = 0.35**	***p* = 0.56**
*Unadjusted intercept*	*14 (12, 16)*		*35 (30, 40)*	
18–49	−3 (−8, 2)	−3 (−7, 1)	5 (−24, 34)	−7 (−27, 14)
50–59	0 (−3, 3)	0 (−3, 3)	4 (−3, 11)	1 (−7, 8)
60–69	Ref	Ref	Ref	Ref
70+	2 (−2, 6)	0 (−3, 2)	12 (−1, 25)	6 (−4, 16)
**Sex**	***p* = 0.11**	***p* = 0.09**	***p* = 0.08**	***p* = 0.93**
*Unadjusted Intercept*	*17 (14, 20)*		*52 (38, 66)*	
Female	Ref	Ref	Ref	Ref
Male	−3 (−7, 1)	−2 (−5, 0)	−13 (−28, 2)	0 (−9, 10)
**Sum of Minor ADGs**	***p* = 0.31**	***p* = 0.10**	***p* = 0.38**	***p* = 0.19**
*Unadjusted Intercept*	*15 (12, 18)*		*47 (34, 60)*	
0–2	Ref	Ref	Ref	Ref
3–4	−2 (−7, 3)	1 (−2, 4)	−12 (−26, 2)	−8 (−16, 1)
5–6	1 (−3, 5)	2 (−1, 5)	−6 (−27, 15)	−6 (−16, 5)
7+	−2 (−5, 1)	−2 (−5, 2)	−6 (−20, 8)	−10 (−20, 0)
**Sum of Major ADGs**	***p* = 0.13**	***p* = 0.09**	***p* = 0.93**	***p* = 0.06**
*Unadjusted Intercept*	*14 (12, 16)*		*39 (32, 46)*	
0	Ref	Ref	Ref	Ref
1	0 (−3, 3)	−1 (−3, 2)	2 (−9, 13)	6 (−3, 14)
2	−1 (−4, 2)	0 (−3, 4)	−3 (−17, 11)	−4 (−13, 4)
3+	7 (0, 14)	6 (0, 12)	4 (−55, 63)	13 (−3, 29)
**Recent Immigration**	***p* = 0.79**	***p* = 0.64**	***p* = 0.97**	***p* = 0.75**
*Unadjusted Intercept*	*14 (13, 15)*		*40 (35, 45)*	
No	Ref	Ref	Ref	Ref
Yes	−1 (−8, 6)	−2 (−8, 5)	−1 (−54, 52)	−3 (−24, 17)
**Material Deprivation**	***p* = 0.04**	***p* = 0.002**	***p* = 0.94**	***p* = 0.09**
*Unadjusted Intercept*	*13 (11, 15)*		*35 (26, 44)*	
1	Ref	Ref	Ref	Ref
2	1 (−3, 5)	−2 (−6, 1)	1 (−15, 17)	−3 (−12, 5)
3	0 (−4, 4)	−2 (−6, 2)	2 (−12, 16)	1 (−10, 12)
4	1 (−3, 5)	0 (−3, 4)	4 (−11, 19)	8 (−3, 19)
5	5 (1, 9)	4 (1, 7)	8 (−11, 27)	10 (−2, 21)
**Rurality**	***p* = 0.38**	***p* = 0.43**	***p* = 0.44**	***p* = 0.61**
*Unadjusted Intercept*	*14 (13, 15)*		*39 (34, 44)*	
Urban	Ref	Ref	Ref	Ref
Rural	2 (−2, 6)	1 (−2, 5)	5 (−8, 18)	3 (−13, 8)
**Histology**	***p* = 0.89**	***p* = 0.76**	***p* = 0.35**	***p* = 0.41**
*Unadjusted Intercept*	*14 (12, 16)*		*39 (34, 44)*	
Adenocarcinoma	Ref	Ref	Ref	Ref
Squamous Cell Carcinoma	1 (−3, 5)	0 (−4, 5)	19 (8, 46)	9 (−5, 23)
Other	0 (−6, 6)	−2 (−8, 4)	10 (−27, 47)	5 (−20, 30)
**Tumor Site**	***p* = 0.30**	***p* = 0.10**	***p* = 0.08**	***p* = 0.36**
*Unadjusted Intercept*	*15 (13, 17)*		*43 (34, 52)*	
Middle Esophagus	1 (−5, 7)	0 (−7, 7)	41 (−2, 84)	−7 (−28, 14)
Lower Esophagus	−1 (−4, 2)	−1 (−3, 2)	−4 (−13, 5)	−4 (−12, 3)
Gastroesophageal Junction	Ref	Ref	Ref	Ref
Other	−7 (−15, 1)	−5 (−9, −1)	−20 (−57, 17)	−19 (−42, −5)
**TCSC**	***p* < 0.0001**	***p* < 0.0001**	***p* = 0.02**	***p* < 0.0001**
*Unadjusted Intercept*	*27 (23, 31)*		*49 (33, 65)*	
01	−17 (−23, −11)	−17 (−23, −11)	−31 (−70, 8)	−23 (−50, 5)
02	−14 (−21, −7)	−17 (−23, −11)	−20 (−41, 1)	−21 (−38, −3)
03	−10 (−16, −4)	−11 (−17, −5)	3 (−42, 48)	7 (−27, 42)
04	−18 (−23, −13)	−17 (−22, 13)	−10 (−27, 7)	−17 (−31, −2)
05	−13 (−23, −3)	−12 (−23, −1)	−29 (−149, 91)	−29 (−114, 55)
06	−18 (−24, −12)	−16 (−21, −11)	−20 (−68, 28)	−17 (−52, 18)
07	−10 (−16, −4)	−10 (−15, −4)	−8 (−27, 11)	−14 (−29, 2)
08	−10 (−25, 5)	−14 (−30, 2)	98 (28, 168)	98 (43, 154)
09	−7 (−20, 6)	−11 (−21, −2)	36 (−12, 84)	41 (−2, 84)
10 *	-	-	-	-
11	−10 (−15, −5)	−10 (−15, −6)	−13 (−32, 6)	−16 (−33, 2)
12	−6 (−16, 5)	−8 (−17, 2)	21 (−67, 109)	−1 (−75, 74)
13	Ref	Ref	Ref	Ref
14	−16 (−22, −10)	−16 (−21, −11)	−22 (−43, −1)	−27 (−43, −11)
15	−14 (−22, −6)	−13 (−21, −6)	−25 (−99, 49)	−31 (−76, 14)
Non−TCSC	−9 (−65, 47)	−13 (−54, 29)	99 (−262, 460)	79 (−77, 234)

* There were no observations in TCSC 10 during the study period from 2013–2018. (CI = confidence interval; ADG = aggregate diagnosis groups; TCSC = thoracic cancer surgery center.)

## Data Availability

The dataset from this study is held securely in coded form at ICES. Although data-sharing agreements prohibit ICES from making the dataset publicly available, access may be granted to those who meet pre-specified criteria for confidential access, available at https://www.ices.on.ca/DAS. The full dataset creation plan and underlying analytic code are available from the authors upon request, understanding that the computer programs might rely on coding templates or macros that are unique to ICES and might therefore either be inaccessible or require modification.

## Supplementary Materials

The following supporting information can be downloaded at: https://www.mdpi.com/article/10.3390/curroncol29080466/s1, Table S1: Database descriptions, Table S2: ICD-O-3 Codes for morphology and topography, Table S3: Codes for diagnosis, consultations, investigations, and treatment, Table S4: Sensitivity analysis comparing TTS in the original model with the inclusion of TTC at the 50th and 90th percentiles.
