# Supplementary material for: Time to Surgery for Patients with Esophageal Cancer Undergoing Trimodal Therapy in Ontario: A Population-Based Cross-Sectional Study
Source: Curr Oncol. 2022 Aug 20;29(8):5901–18. doi: 10.3390/curroncol29080466 (PMC9406364; doi:10.3390/curroncol29080466)
Supplement: Supplementary file 1 [file curroncol-29-00466-s001.zip › Supplementary Table S1.pdf]

Supplementary Table S1

Database	Description
Ontario Cancer Registry	Cancer information, including site, histology, and diagnosis date
Registered Persons Database	Patient demographic data including age, sex, vital status, and dates of last healthcare encounter
Ontario Health Insurance Plan Database	Physician billing database for inpatient and outpatient services, including diagnoses, services provided, and dates
Discharge Abstract Database	Mandatory submissions from hospitals to the Canadian Institute for Health Information, includes information on hospital admission such as dates and diagnoses
Same Day Surgery Database	Stores information such as date and service for same day procedures
National Ambulatory Care Reporting Database	Receives mandatory submissions from institutions for visits made to hospital and community ambulatory care centres
Postal Code Conversion File (PCCF)	Converts a patient's postal code into a dissemination area to ascribe certain characteristics to each patient such as rurality and median household income
Activity Level Reporting	Stores information on chemotherapy and radiotherapy dates and services, both at regional centres and outreach clinics
Local Health Integrated Network (LHIN)	Stores information including population and number / type of hospitals within each LHIN
Ontario Marginalisation	This database comprises separate elements (e.g. material deprivation) and is used in conjunction with PCCF to assign patients a score
IRCC Permanent Resident Database	This includes information on people who applied to land in Ontario such as country of citizenship and date of landing
ICES Physician Database	Demographic information on Ontario physicians including age, specialty, location of work, and year of graduation
