# Supplementary material for: Time to Surgery for Patients with Esophageal Cancer Undergoing Trimodal Therapy in Ontario: A Population-Based Cross-Sectional Study
Source: Curr Oncol. 2022 Aug 20;29(8):5901–18. doi: 10.3390/curroncol29080466 (PMC9406364; doi:10.3390/curroncol29080466)
Supplement: Supplementary file 1 [file curroncol-29-00466-s001.zip › Supplementary Table S2.pdf]

Supplementary Table S2 ICD-O-3 Codes for morphology and topography

**Description**	**Code**
Adenocarcinoma	8140–8141, 8143–8145, 8190–8231, 8260–8263, 8310, 8401, 8480–8490, 8550–8551, 8570–8574, 8576
Squamous Cell Carcinoma	8050–8078, 8083–8084
Other	80001-80003, 80103, 80203, 80223, 80303, 80313, 81482, 81490, 84903, 85603
C15.0	Cervical esophagus
C15.1	Thoracic esophagus
C15.2	Abdominal esophagus
C15.3	Upper third of esophagus
C15.4	Middle hird of esophagus
C15.5	Lower third of esophagus
C15.8	Overlapping lesion of esophagus
C15.9	Esophagus, NOS
C16.0	Cardia, NOS<br>- Gastric cardia<br>- Cardioesophageal junction<br>- Esophagogastric junction<br>Gastroesophageal junction
