# Supplementary material for: Time to Surgery for Patients with Esophageal Cancer Undergoing Trimodal Therapy in Ontario: A Population-Based Cross-Sectional Study
Source: Curr Oncol. 2022 Aug 20;29(8):5901–18. doi: 10.3390/curroncol29080466 (PMC9406364; doi:10.3390/curroncol29080466)
Supplement: Supplementary file 1 [file curroncol-29-00466-s001.zip › Supplementary Table S3.pdf]

**Supplementary Table S3** Codes for diagnosis, consultations, investigations, and treatment. (CT = Computed Tomography; C/A/P = Chest, Abdomen, Pelvis; PET = Positron Emission Tomography; EUS = Endoscopic Ultrasound; PFTs = Pulmonary Function Tests)

| Event | Code | Data Source |
|---|---|---|
| **Biopsy** | 2NA71, 2NC70BN<br>Z515, Z399, +E702<br>150 | CCI<br>OHIP fee<br>OHIP dxcode |
| **Consultations** | | OHIP |
| - Surgery | A643-A646, C643-C646, W645, W646, | |
| - Medical Oncology | A441-A448, A845, C441-C446, C845, W445, W446, W842-W847 | |
| - Radiation Oncology | A340-A348, A745, C341-C346, C745 | |
| **Investigations** | | OHIP |
| - CT (C/A/P) | X125, X406, X407 / X126, X409, X410 / X231, X232, X233 | |
| - CT (head) | X188, X400, X401, X402, +E874 | |
| - PET | J710 | |
| - EUS | S236, E800 | |
| - PFTs | J301, J303, J304, J305, J306, J308, J310, J311, J324, J327, J340, | |
| **Treatment** | | |
| - Endoscopic Resection | S093, Z527, +E674/E675 | OHIP |
| - Chemotherapy | G281, G339, G345, G359, G381, G382 | OHIP |
| - Radiotherapy | 519, 530-542, 548, 549, 575, 592, 594, 596, 597<br>X310-X313 | ALR<br><br>OHIP |
| - Surgery | 1NA87 - 1NA92<br>S089, S090 | CCI<br>OHIP |
