# Supplementary material for: Time to Surgery for Patients with Esophageal Cancer Undergoing Trimodal Therapy in Ontario: A Population-Based Cross-Sectional Study
Source: Curr Oncol. 2022 Aug 20;29(8):5901–18. doi: 10.3390/curroncol29080466 (PMC9406364; doi:10.3390/curroncol29080466)
Supplement: Supplementary file 1 [file curroncol-29-00466-s001.zip › Supplementary Table S4.pdf]

Supplementary Table S4. Sensitivity analysis comparing **TTS** in the original model with the inclusion of TTC at the 50th and 90th percentiles. (CI = Confidence Interval; ADG = Aggregate Diagnosis Groups; TCSC = Thoracic Cancer Surgery Centre)

**Variables**	**Adjusted Differences 50th Percentile (95% CI)**		**Adjusted Differences 90th Percentile (95% CI)**	
	**Original Model**	**Sensitivity Analysis**	**Original Model**	**Sensitivity Analysis**
Adjusted Intercept	*130 (120, 140)*	*119 (109, 130)*	*163 (151, 174)*	*166 (152, 179)*
**Age Group (Years)**	**p = 0.01**	**p = 0.002**	**p = 0.35**	**p = 0.37**
18-49	-11 (-19, -2)	-6 (-15, 2)	-8 (-22, 6)	-11 (-26, 4)
50-59	-1 (-6, 4)	-2 (-7, 3)	-4 (-10, 1)	-4 (-9, 2)
60-69	Ref	Ref	Ref	Ref
70+	4 (-1, 9)	7 (3, 12)	-2 (-7, 2)	-1 (-6, 5)
**Sex**	**p = 0.38**	**p = 0.95**	**p = 0.78**	**p = 0.44**
Female	Ref	Ref	Ref	Ref
Male	-2 (-6, 2)	0 (-4, 5)	-1 (-7, 5)	-2 (-8, 4)
**Sum of Minor ADGs**	**p = 0.40**	**p = 0.65**	**p = 0.86**	**p = 0.39**
			Ref	
0-2	Ref	Ref	-2 (-8, 4)	Ref
3-4	-3 (-9, 2)	0 (-7, 6)	-2 (-8, 4)	-5 (-11, 1)
5-6	1 (-4, 6)	3 (-3, 9)	-2 (-8, 4)	-5 (-12, 2)
7+	-2 (-8, 4)	1 (-6, 7)		-3 (-10, 3)
**Sum of Major ADGs**	**p = 0.41**	**p = 0.49**	**p = 0.61**	**p = 0.49**
0	Ref	Ref	Ref	Ref
1	1 (-4, 5)	0 (-5, 5)	3 (-2, 8)	2 (-3, 8)
2	4 (-1, 9)	3 (-3, 9)	3 (-4, 9)	1 (-5, 8)
3+	4 (-4, 11)	-3 (-11, 5)	3 (-5, 12)	7 (-3, 17)
**Material Deprivation**	**p = 0.32**	**p = 0.03**	**p = 0.21**	**p = 0.91**
1	Ref	Ref	Ref	Ref
2	5 (-1, 12)	11 (4, 18)	-1 (-7, 5)	-3 (-10, 4)
3	4 (-2, 10)	7 (1, 13)	1 (-5, 7)	-0 (-7, 6)
4	7 (1, 14)	9 (2, 16)	1 (-6, 7)	-1 (-8, 6)
5	7 (0, 14)	8 (2, 15)	2 (-5, 9)	1 (-8, 9)
**Rurality**	**p = 0.68**	**p = 0.81**	**p = 0.25**	**p = 0.12**
Urban	Ref	Ref	Ref	Ref
Rural	3 (-3, 8)	1 (-5, 6)	3 (-2, 9)	5 (-1, 11)

Variables	Adjusted Differences 50th Percentile (95% CI)		Adjusted Differences 90th Percentile (95% CI)	
	Original Model	Sensitivity Analysis	Original Model	Sensitivity Analysis
**Recent Immigration**	**p = 0.19**	**p = 0.41**	**p = 0.92**	**p = 0.24**
No	Ref	Ref	Ref	Ref
Yes	2 (-8, 12)	4 (-5, 13)	8 (-6, 22)	11 (-7, 29)
**Histology**	**p = 0.06**	**p = 0.24**	**p = 0.72**	**p = 0.98**
Adenocarcinoma	Ref	Ref	Ref	Ref
Squamous Cell Carcinoma	-5 (-11, 1)	-5 (-11, 2)	3 (-4, 9)	0 (-8, 7)
Other	10 (-3, 22)	5 (-7, 18)	2 (-12, 17)	-1 (-18, 15)
**Tumour Site**	**p = 0.68**	**p = 0.79**	**p = 0.93**	**p = 0.76**
Middle Esophagus	4 (-6, 15)	6 (-6, 18)	1 (-8, 10)	0 (-12, 11)
Lower Esophagus	0 (-4, 4)	1 (-4, 5)	0 (-5, 4)	1 (-4, 6)
Gastroesophageal Junction	Ref	Ref	Ref	Ref
Other	-6 (-22, 10)	2 (-13, 17)	-4 (-20, 12)	-6 (-21, 9)
**TCSC**	**p <.0001**	**p <.0001**	**p = 0.003**	**p = 0.10**
01	19 (3, 35)	21 (4, 38)	13 (-6, 32)	15 (-5, 35)
02	19 (11, 28)	21 (13, 30)	13 (3, 22)	12 (1, 23)
03	-5 (-12, 3)	-2 (-11, 8)	-4 (-16, 8)	0 (-16, 16)
04	14 (5, 22)	15 (7, 24)	13 (5, 21)	12 (4, 21)
05	-8 (-28, 12)	-11 (-40, 17)	7 (-19, 33)	9 (-33, 51)
06	5 (-4, 14)	4 (-6, 14)	7 (-8, 23)	8 (-6, 22)
07	3 (-5, 11)	4 (-4, 13)	1 (-10, 11)	1 (-10, 11)
08	16 (5, 28)	19 (6, 31)	11 (-10, 32)	11 (-17, 38)
09	-13 (-33, 7)	-9 (-28, 9)	4 (-22, 30)	6 (-23, 35)
10	-	-	-	-
11	4 (-8, 16)	8 (-3, 20)	12 (3, 21)	11 (2, 21)
12	19 (10, 27)	26 (15, 37)	10 (-1, 21)	11 (-8, 31)
13	Ref	Ref	Ref	Ref
14	-4 (-13, 4)	-1 (-10, 8)	0 (-17, 17)	3 (-14, 21)
15	7 (-7, 21)	1 (-14, 17)	9 (-13, 31)	10 (-19, 39)
Non-TCSC	7 (-12, 26)	-3 (-23, 18)	-9 (-64, 47)	1 (-102, 104)
